# Wild edible plant species utilized by a subsistence farming community in Obalanga sub-county, Amuria district, Uganda

**DOI:** 10.1186/1746-4269-11-7

**Published:** 2015-02-10

**Authors:** Samuel Ojelel, Esezah K Kakudidi

**Affiliations:** Department of Biological Science, Makerere University, College of Natural Science, P.O. Box 7062, Kampala, Uganda

**Keywords:** Wild edible plants, Indigenous knowledge, Subsistence farming, Consumption

## Abstract

**Background:**

Farming communities have continuous interactions with their environment. Subsistence farmers are particularly vulnerable to the vagaries of weather. These are pre-requisites for increased wild edible plant consumption. This study mainly focused on indigenous knowledge regarding identity and use of wild edible plant species by the subsistence farmers of Obalanga.

**Methods:**

A multistage sampling technique was used to identify Agonga parish. Systematic random sampling was used to locate 64 respondents stratified among children, adult females and males. After obtaining informed consent and assent, data was collected through semi-structured interviews using a checklist of open ended questions, focus group discussions and guided field visits. The free listing technique was employed to obtain data on plant identity and usage.

**Results:**

Fifty one (51) species in forty three (43) genera spread in thirty two (32) families were identified. Age and gender had significant effects on respondents’ wild edible plant species knowledge. The majority of edible wild plant species were herbs (47.1%) while grasses (3.9%) were the least. Fruits (51.0%) were the major parts consumed while tubers and roots constituted only 2.0% each. Eating uncooked as snacks (43.1%) was the favoured mode of consumption compared to roasting (2.0%). Preservation was mainly by solar drying. Wild edible plants traded within and without Obalanga community constituted only 15.7%. Almost all the edible plant species (94.1%) do not have any specific bye-laws for their conservation. Only *Mangifera indica, Tamarindus indica* and *Vittaleria paradoxa* representing 5.9% of the species are protected by bye-laws.

**Conclusion:**

Disproportionate distribution of edible wild plant indigenous knowledge was noted in Obalanga with the lowest among the children. The marketed plant species in Obalanga can offer an opportunity for household livelihood diversification through value addition and trade under the umbrella of organic products. This will increase household incomes thereby contributing towards MDG 1 on eradicating extreme poverty and hunger. It is thus vital to document indigenous knowledge so that it is not lost as plant species disappear due to environmental degradation.

## Introduction

Wild edible plants have formed part of human diet since time immemorial with nearly 75,000 species of plants believed to be edible [1–4]. It is estimated that humans have domesticated about 200 species as food crops but around 30 only contribute 95% of the world’s plant food intake [5, 6]. However, despite the primary reliance of agricultural communities on conventional crop plants, the tradition of eating wild plants has never disappeared [7–9].

In Africa, many communities still gather and consume edible wild plants [10, 11]. Rural communities use these plants to supplement their diets which are based on a narrow range of rain-fed staples [12]. It is commonly reported that consumption of these plants is particularly vital at times of food shortage because they enhance livelihoods, survival strategies and support household economies [12–15]. Their importance is exemplified by free and easy accessibility and nutritional richness especially vitamins and micronutrients [12, 16–18]. Therefore, they play a significant role in the livelihoods of rural communities through improved household incomes and food security [19, 20]. Indeed, FAO in its state of food insecurity in the world report estimated that around one billion people use wild plants in their diet [21].

In Uganda, research on wild edible plants has received significant attention from various researchers [9, 22–25]. However, FAO stressed that the conservation and promotion of sustainable utilization of neglected food plants requires various actions including inventorying, *in situ* conservation of wild relatives and promotion of development and commercialization [21]. Similarly, many values from these plants remain undocumented because their products are used locally without being reflected in national or international markets [6, 26, 27]. Therefore, systmatic documentation of indigenous knowledge regarding the identity and use of wild foods by rural communities has become an urgent concern because both biological resources and indigenous knowledge are diminshing with hatitat destruction and a growing disinterest among the younger generation [28]. It is on this background that this sudy sought to (i) inventory wild edible plants (ii) determine the preparation, preservation, marketability and conservation and (iii) determine the influence of age and gender on the knowability of edible wild plant species among the subsistence farmers of Obalanga, Amuria district, Uganda.

## Methods and materials

### Study area

Obalanga sub-county is located in Amuria district in the North Eastern part of Uganda. It lies between Latitude 33^°^30’E to 33^°^45’E and Longitudes 2^°^24’N to 2^°^45’N. The community comprises mainly Ateso speaking people. They are subsistence farmers growing cereals such as millet, sorghum; legumes like cowpeas, groundnuts, green grams and tubers like cassava, sweet potatoes on rain-fed basis. The soils are mainly ferralytics. They also rear livestock such as cattle, goats and sheep. The vegetation cover is predominantly savannah dominated by *Combretum* species and *Vittaleria paradoxa.*

### Sample selection and data collection

A multistage sampling technique was used to select Agonga parish. In this technique, Obalanga sub county was randonly selected from the nine sub-counties of Amuria district as the Primary Sampling Unit (PSU). Within Obalanga sub-county, Agonga parish was randomly selected as the Secondary Sampling Unit (SSU) and thereafter all the eight villages (Aguyaguya, Ajesai, Amoni, Aridai, Karamata, Ocongoda, Okerai and Ongopai) therein were considered as the Ultimate Sampling Units (USU) where the respondents were located. Sixty four respondents stratified into 24 children (below 18 years), 22 adult females (>18 years) and 18 adult males (>18 years) were interviewed. Stratification enabled effective representation of the respondents along age and gender differences. At least two respondents in each stratum and six from each village were interviewed. These respondents were located using systematic random sampling whereby the first respondent was randomly picked upon reaching a new village and thereafter respondents in every third household were considered until the required number was attained. Only a single respondent was interviewed from each household. Informed consent and assent for the case of children was obtained from their parents and guardians. Data was collected through semi-structured interviews using a checklist of open ended questions. Since the data required was mainly indigenous knowledge pertaining to edible plant identity and use, the free listing technique was used. In this technique, the respondents were asked to mention any plant that comes to their mind until they could not mention any more species. It is generally agreed that normally people remember plants which are important to them [29]. Field guided walks to the farm lands, grazing lands and other open habitats were led by the respondents to collect the plants listed during the interiew. This process was not repeated if the respondent mentioned plants whose specimens had already been collected. Two Focus Group Discussions (FGDs) were held in Amoni and Aridai villages to authenticate the data in questionnaires and capture additional responses. All the voucher plant specimes were identified at Makerere University Herbarium.

### Data analysis

The Familiarity Index (FI) to exlain the importance of each species was calculated directly from the number of respondents who mentioned the species across the strata [14]. The responses on use were tallied and trends presented using graphs. A non-parametric spearman rank correlation test in SPPS ver. 16.0 was used to examine the influence of age and gender on the individuals wild edible plant knowledge basing on their FIs.

## Results and discussion

### Wild edible plant diversity

Fifty one (51) species in 43 genera belonging to 32 families were documented (Table 1). The family Malvaceae had the largest number of species followed by Anarcadiaceae, Moraceae and Solanaceae. *V. paradoxa, M. indica* and *T. indica* were the most mentioned edible plant species in Obalanga (Table 1). *M. indica* has many varieties but are not reported in this study. The diversity of species affirms that edible wild plants consumption among the subsistence farmers in Obalanga is still active and is important in their daily plant food intake. It also shows that the environment is diverse and great interaction currently exists. The high popularity of *V. paradoxa, M. indica* and *T. indica* indicate their perceived economic and traditional significance to households. *V. paradoxa* in particular enjoys cultural acceptability and enjoyment among the Iteso community as smearing oil for new borne babies. The availability, palatability and multipurpose nature of these species also make them more popular than others among the community [30]. Some of the species with very low FIs (Table 1) such as *H. opposita* was unknown to children suggesting that it is either scarce, only consumed during times of food shortages or currently neglected. It has also been regularly documented that areas with recurrent food shortages tend to rely on edible wild plants [13, 15]. This observation explains the prevailing situation in Obalanga because the area has undergone recurrent extreme weather conditions (drought and floods) and armed cattle rustling by the neighbouring Karamojongs. With the escalating effects of climate change, wild edible plants could play a significant role in rural household coping and adaptation strategies since they have greater adaptability than the conventional food crops.

**Table 1:** **Wild edible plants consumed in Obalanga**

Family	Species name and Voucher numbers	Genus	Local name	Ha	PC	MC	FI	MKT
Acanthaceae	*Asystasia mysorensis (Roth.) T. Anderson (OS-002)*	Asystasia	*ecototo*	H	L	Co	26.6	NM
Amaranthaceae	*Amaranthus dubius Mart. ex. Thell (OS-015)*	Amaranthus	*aboga*	H	Sho	Co	3.1	M
Anacardiaceae	*Mangifera indica L. (OS-021)*	Mangifera	*emiebe*	T	F	Uc	88.4	M
	*Rhus natalensis Bernh. (OS-039)*	Rhus	*ewayo*	T	F	Uc	43.4	NM
	*Sclerocarya birrea Hochst. (OS-001)*	Sclerocarya	*ejikai*	T	F	Uc	43.8	NM
Annonaceae	*Annona senegalensis Pers. (OS-042)*	Annona	*ebwolo*	T	F	Uc	35.9	NM
Apocynaceae	*Mondia whitei (Hook.f.) Skeels (OS-025)*	Mondia	*emulondo*	H	R	Uc	1.6	M
Arecaceae	*Borassus aethiopum Mart. (OS-014)*	Borassus	*edukudukut*	T	F	Uc	10.9	NM
Asteraceae	*Bidens pilosa L. (OS-043)*	Bidens	*enyikibon*	H	L	Co	1.6	NM
Commelinaceae	*Commelina africana L. var. africana (OS-016)*	Commelina	*ekoropot*	H	Sho	Co	6.3	NM
Convolvulaceae	*Ipomoea eriocarpa R.Br. (OS-045)*	Ipomoea	*eriono*	H	L	Co	3.1	NM
Ebenaceae	*Diospyros mesipiliformis Hochst. ex A. DC. (OS-003)*	Diospyros	*ekum*	T	F	Uc	9.4	NM
Euphorbiaceae	*Acalypha lanceolata Willd. (OS-044)*	Acalypha L.	*ekiliton*	H	Sho	Co	10.9	NM
Fabaceae	*Cassia obtusifolia L. (OS-050)*	Cassia	*eedo*	H	L	Co	29.7	NM
	*Tamarindus indica L. (OS-012)*	Tamarindus	*epeduru*	T	F/L	Uc/Co	70.3	M
	*Abrus precatorius L. subsp. africanus Verdc. (OS-024)*	Abrus	*ewinyiwiny*	L	L	Uc	1.6	NM
	*Eriosema shirense Baker f. (OS-040)*	Eriosema	*adokolet*	G	Tu	Uc/Ro	6.3	NM
Hypoxidaceae	*Hypoxis rigidula Baker. (OS-049)*	Hypoxis	*ekiomi*	H	F	Uc	4.5	NM
Lamiaceae	*Leonotis nepetifolia (L.) R. Br. (OS-013)*	Leonotis	*ajon icika*	H	F	Uc	1.6	NM
	*Hoslundia opposita Vahl. (OS-023)*	Hoslundia	*etuutuu*	H	F	Uc	1.6	NM
	*Vitex doniana Sweet. (OS-027)*	Vitex	*eelu*	T	F	Uc	6.3	NM
	*Vitex madiensis Oliv. (OS-041)*	Vitex	*ekwarukei*	T	F	Uc	39.1	NM
Loganiaceae	*Strychnos innocua Delile (OS-004)*	Strychnos	*ekwakwalet*	T	F	Uc	26.6	NM
Malvaceae	*Grewia mollis Juss. (OS-046)*	Grewia	*eparis*	T	F	Uc	42.2	NM
	*Hibiscus aculeatus Walter (OS-026)*	Hibiscus	*egwanyira*	H	L	Co	25	NM
	*Corchorus trilocularis L. (OS-047)*	Corchorus	*etigo-apio*	H	L	Co	45.3	NM
	*Corchorus aestuans L.(OS-022)*	Corchorus	*etigo-akolocoro*	H	L	Co	31.3	NM
	*Corchorus sp. (OS-006)*	Corchorus	*etigo-aar ikiliok*	H	L	Co	1.6	NM
Moraceae	*Ficus platyphylla Delile. (OS-048)*	Ficus	*ebule*	T	F	Uc/Co	7.8	NM
	*Ficus glumosa Delile (OS-051)*	Ficus	*ebyong*	T	F	Co	6.3	NM
	*Ficus natalensis Hochst. (OS-005)*	Ficus	*ecalonyi*	T	F	Co	7.8	NM
	*Ficus sur Forssk. (OS-007)*	Ficus	*eboborei*	T	F	Uc/Co	4.7	NM
Nymphaeaceae	*Nymphaea lotus L. (OS-018)*	Nymphaea	*ekoromit*	H	F	Uc	4.7	NM
Pedaliaceae	*Sesasum angutifolium (Oliv.) Engl. (OS-028)*	Sesasum	*emelerait*	H	L	Co	26.6	NM
Phyllanthaceae	*Bridelia scleronuera Müll.Arg. (OS-037)*	Bridelia	*erieco*	T	F	Uc	40.6	NM
Poaceae	*Cymbopogon citratus (DC.) Stapf. (OS-032)*	Cymbopogon	*enyait*	G	Sho	Co	1.6	NM
Polygonaceae	*Polygonum pulchrum Blume. (OS-011)*	Polygonum	*dekerngude*	H	L	Co	1.6	NM
	*Polygonum sinuatum Royle ex Bab. (OS-035)*	Polygonum	*esugugur*	H	Sho	Co	1.6	NM
Portulacaceae	*Portulaca oleracea L. (OS-019)*	Portulaca	*etebire*	H	Sho	Co	3.1	NM
Rhamnaceae	*Ziziphus abyssinica Hochst. (OS-008)*	Ziziphus	*emuriei*	S	F	Uc	64.5	NM
Rubiaceae	*Sarcocephalus latifolius (Sm.) Bruce (OS-031)*	Sarcocephalus	*eutudolei*	T	F	Uc	12.5	NM
Sapotaceae	*Vittelaria paradoxa C.F. Gaertn (OS-036)*	Vittelaria	*ekungur*	T	F/Se	Uc/Co	87.5	M
Solanaceae	*Capsicum frutescens L. (OS-010)*	Capsicum	*emulalu*	Sh	L/F	Uc/Co	15.6	M
	*Physalis peruviana L. (OS-033)*	Physalis	*etagoli loapolon*	H	F	Uc	6.3	NM
	*Physalis minima L. (OS-020)*	Physalis	*etagoli lodidi*	H	F	Uc	3.1	NM
	*Lycopersicon esculentum Mill. (OS-030)*	Solanum	*esalamejei*	H	F	Co	45.3	M
Verbenaceae	*Lantana camara L. (OS-034)*	Lantana	*elatana*	Sh	F	Uc	4.7	NM
Vitaceae	*Cyphostemma adenocuale (Steud.ex A.Rich) Desc.ex Wild R.B. Drumm. (OS-009)*	Cyphostemma	*Emoros*	L	L	Uc/Co	37.5	NM
Ximeniaceae	*Ximenia americana L.(OS-038)*	Ximenia	*elamai*	T	F/L	Uc/Co	60.1	NM
Zingiberaceae	*Afromomum alboviolacuem (Ridl.) K. Schum (OS-029)*	Afromomum	*acawoi*	H	F	Uc	20.3	NM
Balanitaceae	*Balanites aegyptiaca (L.) Delile. (OS-017)*	Balanites	*ecomai*	T	F/L	Uc/Co	51.6	M

Key: Ha (Plant habit): H=Herb, G=Grass, Sh=Shrub, T=Tree and L=Liana. MC (Mode of Consumption): Co=Cooked, Uc=Uncooked, Co/Uc=Cooked and/or Uncooked, Uc/Ro-Uncooked and/or Roasted. PC (Plant parts consumed): Sho=Shoot, F=Fruit, Se=Seed, Tu=Tuber, Le=Leaf, R=Root and FI=Familiarity Index. MKT (Local Marketability): NM=Non Marketed, M=Marketed.

The diversity of wild edible plants in Obalanga subcounty compares closely with findings of earlier investigators on the African and Asian continent: For instance; 41 species belonging to 17 families in the Manang District of Central Nepal, 67 species belonging to 30 families in the Nhema communual area, Midlands province of Zimbabwe, 66 species in 34 families in Derashe and Kucha districts of South Ethiopia, 57 species in 33 families among the Gujjar tribe of India and 62 species in 31 families in Bunyoro-Kitara, Uganda [7, 9, 12, 31, 32]. All these investigators attribute the diversity of the wild edible plants in the respetive communities to the diversity of plant species within the surrounding environment and subsequently the rich indigenous knowledge.

### Growth habits

**Figure 1:**
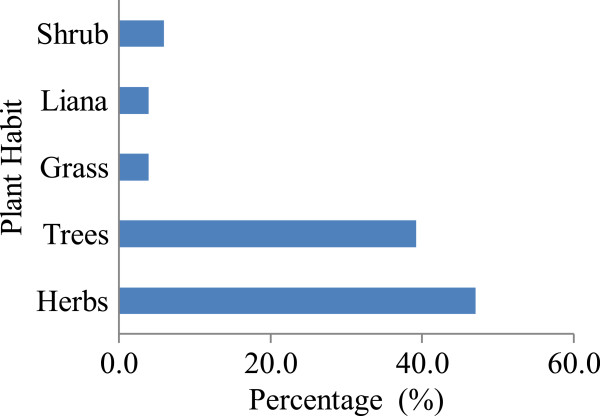
**Growths habits of wild edible plants in Obalanga.**

Herbaceous species comprise the majority of edible wild plants (47.1%) consumed in Obalanga subcounty. This is closely followed by trees (39.2%) while grasses and lianas at 3.9% each are the least (Figure 1). The ephemerous nature of herbs makes them available during most months of the year within the gardens, grazing lands and around homesteads. This pattern was also reported in the Gujjar tribe of Rajouri in Jammu and Kashmir state of India; the Bunyoro-Kitara kingdom of Uganda and Nhema Communual area, Midlands province of Zimbabwe [9, 12, 32]. The trend has been attributed to the diversity of herbaceous plants in the surrounding environment [33]. All these factors ably explain the current status among the subsistence farmers of Obalanga subcounty. They also have a higher tolerance to weather changes as compared to conventional staples.

### Plant parts consumed

**Figure 2:**
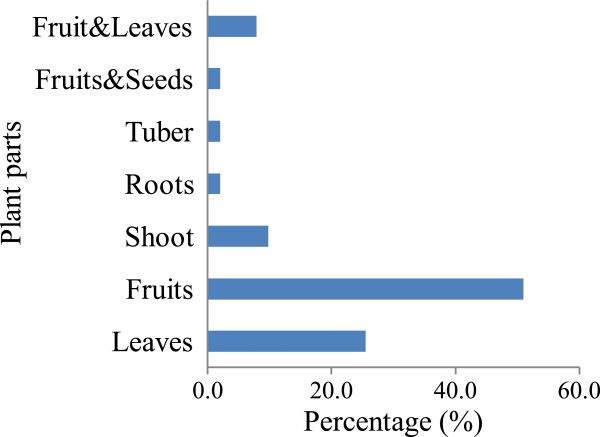
**Wild edible plant parts consumed in Obalanga.**

The major parts consumed in Obalanga subcounty are shown in Figure 2. Fruits (51.0%) were the major plant parts consumed. In Balumogi County of Uganda, in Nhema communual area, Zimbabwe and around Kibale National Park (Uganda), a similar trend has been reported [12, 14, 25]. The major determining factor is the amount of effort (labour) required in preparing them. Only *V. paradoxa* seeds were mentioned as edible. However, during the focus group discussions, respondents mentioned that some of the seeds are actually eaten together with the fruits for instance *C. frutescens, P. peruviana* and *P. minima.* This is because the fruits are small in size and removing seeds may damage the whole fruit and they do not pose any danger to the consumer. In the present study, respondents also reported eating some plants for medicinal purposes for instance, *C. frutescens* fruits are swallowed to remove intestinal worms, the leaves of *Cyphostemma adenocuale* are chewed after one has eaten sour foods.

### Marketability

The wild edible plants marketed within and without Obalanga constitute 15.7%. During this study, a plastic mug of *V. paradoxa* seeds cost between USD 0.2-0.4. This confirms that some wild edible plants contribute not only to the household food basket but also to their incomes. However, it has been reported that most products are eaten directly within the household thereby making it hard to capture the quantity and diversity of the harvest at local or national level [34]. This observation represents the trade in wild edible plants in Obalanga. Plants such as *S. birrea* have potential use in the wine industry but is neither exploited nor marketed in Obalanga. There is however no doubt that the marketable edible wild plants have the potential to enhance household incomes once fully tapped. This can have a contributary role towards attainment of the MDG 1 on eradicating extreme poverty and hunger.

### Modes of consumption

**Figure 3:**
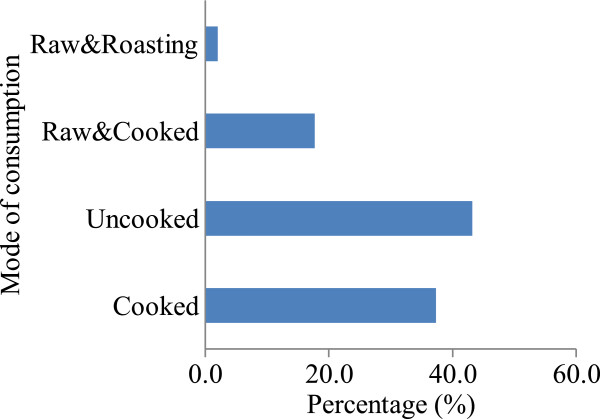
**Modes of wild edible plant consumption in Obalanga.**

The major consumption modes are shown in Figure 3. Non-cooked eating (43.1%) in the form of snacks ranks highest. The popular species in this category include among others *A. albovialaceum, S. birrea, Z. abyssinica* (Table 1). Uncooked eating as snacks arises during hours spent away from home or carried and eaten at home. The majority of such items are eaten instantly from collection areas such as grazing land, farmland and road sides. Plants such as *T. indica* and *A. albovialaceum* fruits can be locally processed and drunk as juice singly or in combination with other foods such as cooked sweet potatoes. In Nhema communual area of Zimbabwe and Derashe and Kucha districts of South Ethiopia, high consumption in the form of snacks is attributable to easiness of processing, perceived high nutritional value and the desirable taste [7, 12].

### Modes of preparation

The plants that require cooking (37.3%) shown in Figure 3 undergo some kind of preparation. *B. aethiopum, T. indica* and *S. innocua* that are eaten as snack require specialized preparation. For instance; the fruits of *B. aethiopum* are collected, taken to a nearby hard surface (such as rock or road) and hit on the ground several times until it softens and cracks appear, the fruit is then washed and is ready for eating. As for the *B. aegyptiaca*, residents summarize the process as follows: Cut the branch, hand pick the leaves, wash, pound in a mortar with pestle then boil. Sour milk or groundnut paste is added but no salt and the sauce should be eaten while warm to prevent hardening. The rest of the plants eaten as snacks need just washing or most often eaten without washing. The cutting of branches of *B. aegyptiaca* is destructive to the plant and removes mature branches that would produce fruits during the next season. Thus, this kind of harvesting reduces the productivity of the trees.

### Preservation methods

All the plants cooked are also preserved (Figure 2). Only four plants which are eaten as snacks can be preserved namely *T. indica, V. paradoxa, C. frutescens* and *M. whitei.* For instance, the dry fruits of *T. indica* can be preserved with or without their pericarps. The former offers better hygiene while the latter eases detection of weavil invasion. *C. frutescens* can be preserved with or without the branches. The former is prefered to the latter because it can be easily suspended from the roof of the house or kitchen beyond the reach of children. The primary preservation technique in Obalanga is direct solar drying. Storage containers include guords, polythene bags and clay pots. Some are also kept together with agricultural produce in the granaries. Preservation and storage is mainly a coping strategy to bolster availablity during periods of scarcity for instance during the dry season between November and March. The perishability of the plant parts consumed determine whether or not they are preserved. For instance, although the communities would wish *M. indica* to be available during periods of scarcity, limited knowledge on preservation remains a major hinderance. Preservation would have an important role in sustaining households in periods of human and nature induced calamities like armed conflict and drought respectively. It can also ensure that the plants are marketed beyond the harvest season. There is thus need for adoption of improved preservation techniques if products are to be traded at national or even international markets.

### Influence of age and gender on knowledge of respondents on wild edible plant species

**Figure 4:**
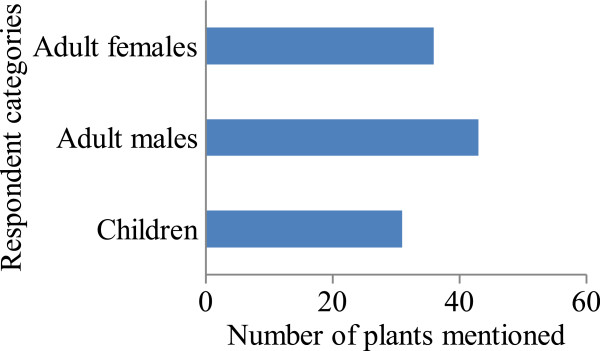
**Influence of sex and gender on wild edible plant knowledge in Obalanga.**

Figure 4 shows the influence of age and gender on the respondents’ wild edible plant knowledge. A non-parametric Spearman rank correlation test showed that gender has a significant effect on the individual’s knowledge (rho = 0.608, p = 0.001, N = 51). This demonstrates that knowledge held is directly related to the responsibilities assigned to or performed by an individual in the community. For instance, adult females were knowledgeable on plants that are cooked while the adult males were knowledgeable on those that are eaten as snacks. This shows that adult females are responsible for cooking in the families while the males who spend most of the time grazing animals, hunting are familiar with snacks. Children’s knowledge also differed significantly from that of adult females (rho = 0.795, p = 0.001, N = 51) and adult males (rho = 0.727, p = 0.001, N = 51). In Figure 4, 20 species from the 51 recorded in Obalanga were not mentioned by children. Focus Group discussions showed that some species such as *S. latifolius, F. sur* were only consumed during periods of extreme food shortage and thus are unknown to children. This trend has also been attributed to low accessibility of the plants [28]. This unequal distribution of indigenous knowledge has been reported in Western Uganda mainly due to the low interest among the young generation to learn about wild edible plants and the universal primary and secondary education which has limited their time of interaction with their families and the natural environment [9]. When the children lose interest at an early age, it is seldom that they will get interested when they grow older. This situation is exacerbated by the loss of edible wild plants indigenous knowledge with increasing plant scarcity [35]. However, it is also worth highlighting that some scholars have reported indigenous knowledge to increase with individuals’ age [36]. This latter finding would ably apply to Obalanga if rapid habitat loss and change was not eminent. There is therefore a need to systematically document this indigenous knowledge so that is does not disappear simultaneously with plants. In this way, it can be preserved for future generations and thereby maintain the local as well as national sovereignty.

### Conservation measures

The conservation measures recorded in Obalanga subcounty include agro-forestry; trees around homesteads, schools and churches, protection from fire and regulation of cutting*.* Conservation of *T. indica, M. indica* and *V. paradoxa* representing 5.9% of all the species is supported by community bye-laws and ancestral land tenure systems. The multi-purpose benefits have been shown to attract greater conservation measures [30]. The herbs which constitute the greatest lifeforms consumed (Figure 2) are mainly seasonal crops and are perceived as readily available and thus do not need specialized conservation measures. Some of them like *B. pilosa* are regarded as weeds once found on farmlands. It has been widely reported that when people repeatedly exploit a species in various ways, the value of that species will be reinforced [6, 37]. This explains the presence of bye-laws for the protection of three selected tree species in Obalanga. The multiple uses and the consumptive value also justify conservation measures geared towards these selected species [12, 14]. Therefore, the social and economic benefits of species generated community interest in them leading to their conservation [38]. This conlusion neglects species with little known values and yet they could become very useful with increase in our knowledge and technology. Prioritization of species in conservation therefore needs to expand beyond just their present social and economic values.

### Conclusion

Disproportionate distribution of edible wild plant indigenous knowledge was noted within the farming community of Obalanga. This finding underlies the importance of developing comprehensive storage and dissemination facilities for this knowledge so that it is not lost as plants become scarce due to environmental degradation. This study has recorded 51 wild edible plants in 43 genera and 32 families. The plants that are marketed within and without Obalanga can offer an opportunity for household poverty alleviation through value addition and trade under organic products umbrella. The consumption of wild plants as food and medicine purposes has also been documented. The greater the benefits from a plant, the higher the prioritization of such a plant in conservation endeavours by the community. Future considerations could focus on the nutraceutical value, income enhancement through value addition and possibility of deliberate management of selected species such as *A. albovialacuem*. This will have a vital role in highlighting the value of wild edible plants to household incomes and food security thereby bolstering efforts to attain MDG 1 on eradicating extreme poverty and hunger and coping with the effects of climate change.
